# Survival prediction in triple negative breast cancer using multiple instance learning of histopathological images

**DOI:** 10.1038/s41598-022-18647-1

**Published:** 2022-08-25

**Authors:** Piumi Sandarenu, Ewan K. A. Millar, Yang Song, Lois Browne, Julia Beretov, Jodi Lynch, Peter H. Graham, Jitendra Jonnagaddala, Nicholas Hawkins, Junzhou Huang, Erik Meijering

**Affiliations:** 1 School of Computer Science and Engineering, UNSW Sydney, Kensington, NSW 2052 Australia; 2 Department of Anatomical Pathology, NSW Health Pathology, St. George Hospital, Kogarah, NSW 2217 Australia; 3 St. George and Sutherland Clinical School, UNSW Sydney, Kensington, NSW 2052 Australia; 4 Faculty of Medicine and Health Sciences, Sydney Western University, Campbelltown, NSW 2560 Australia; 5 University of Technology Sydney, Ultimo, NSW 2007 Australia; 6 Cancer Care Centre, St. George Hospital, Kogarah, NSW 2217 Australia; 7 School of Population Health, UNSW Sydney, Kensington, NSW 2052 Australia; 8 School of Medical Sciences, UNSW Sydney, Kensington, NSW 2052 Australia; 9 University of Texas at Arlington, Arlington, TX 76019 USA

**Keywords:** Breast cancer, Biomedical engineering

## Abstract

Computational pathology is a rapidly expanding area for research due to the current global transformation of histopathology through the adoption of digital workflows. Survival prediction of breast cancer patients is an important task that currently depends on histopathology assessment of cancer morphological features, immunohistochemical biomarker expression and patient clinical findings. To facilitate the manual process of survival risk prediction, we developed a computational pathology framework for survival prediction using digitally scanned haematoxylin and eosin-stained tissue microarray images of clinically aggressive triple negative breast cancer. Our results show that the model can produce an average concordance index of 0.616. Our model predictions are analysed for independent prognostic significance in univariate analysis (hazard ratio = 3.12, 95% confidence interval [1.69,5.75], *p* < 0.005) and multivariate analysis using clinicopathological data (hazard ratio = 2.68, 95% confidence interval [1.44,4.99], *p* < 0.005). Through qualitative analysis of heatmaps generated from our model, an expert pathologist is able to associate tissue features highlighted in the attention heatmaps of high-risk predictions with morphological features associated with more aggressive behaviour such as low levels of tumour infiltrating lymphocytes, stroma rich tissues and high-grade invasive carcinoma, providing explainability of our method for triple negative breast cancer.

## Introduction

Breast cancer is the most prevalent type of cancer and the leading cause of cancer deaths worldwide, with more than 2.2 million cases diagnosed and over 680 thousand breast cancer deaths reported globally in 2020^1^. Current routine histopathology practice analyses key tumour morphological features to provide important information to guide treatment decisions. Histological subtype, grade, tumour size, tumour infiltrating lymphocytes (TILs) density, and lymph nodal status are important prognostic variables supplemented by four biomarkers: estrogen receptor (ER), progesterone receptor (PR), human epidermal growth factor receptor-2 (HER2) and Ki67 which are largely unchanged in almost 20 years.

Our research is focused on triple negative breast cancer (TNBC), defined by the absence of expression of ER and PR along with absence of amplification of the HER2 gene^2^. It is an aggressive type of breast cancer accounting for about 10–20% of all breast cancers^3^. Both overall survival and disease-specific survival of TNBC patients are worse compared to non-TNBC patients with a 5-year survival of 60–70%. TNBC is also more common among younger patients and may be associated with breast invasive carcinoma (BRCA) mutations and familial inheritance, making it a particularly significant type of cancer. Although 5-year survival of TNBC patients is generally poor, those patients who survive beyond this have an excellent prognosis. However, the ability to predict those patients with favourable outcome is currently limited. Traditionally, risk assessment for TNBC is based on clinicopathological parameters visually assessed by pathologists. Treating oncologists use these features to estimate risk of recurrence and guide treatment decisions supplemented by the use of online clinical algorithms^4–6^. To make the decision-making process less labour intensive and faster, it would be desirable to develop a system that can rapidly and objectively interrogate tumour features with equal performance to an expert pathologist while maintaining universal applicability on any data cohort regardless of region of origin, equipment used, or level of expertise of the observer.

**Figure 1:**
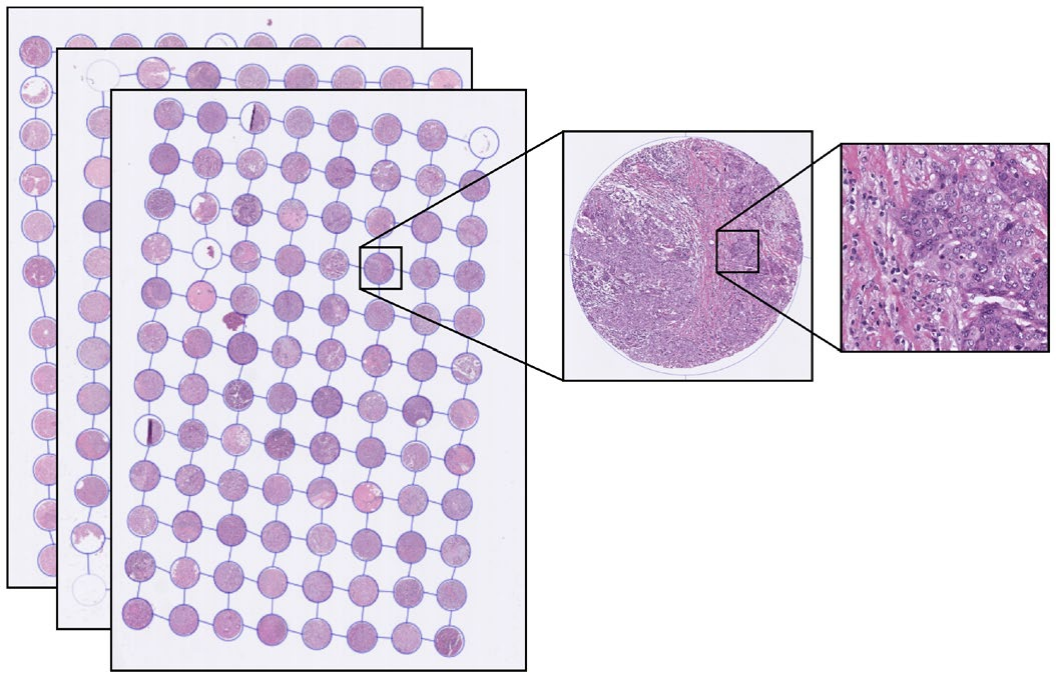
Magnified view of a tissue patch (right) extracted from one core (middle) of a TMA (left) from the TNBC cohort. On average each core is 1.25 mm in diameter. All slides were scanned at 0.25 µm/pixel resolution.

Deep learning has gained recognition as a method of developing fast and accurate computational models to perform complex real-world tasks at the same level of performance as an expert human in that field. It is particularly transformative in the field of computational pathology, where large datasets and resource intensive annotation processes are two common challenges^7–12^. Recently, deep learning models are used successfully in trying to predict risk and survival for various cancer types. A convolutional neural network (CNN)^13^ with convolutional and fully connected layers was used for survival prediction in low-grade glioma (LGG) and glioblastoma multiforme (GBM) and was able to obtain a concordance index (c-index) of 0.741. PAGE-Net^14^ used an architecture with a CNN pathway for histopathological images and a separate fully connected pathway for transcriptomic data to produce a c-index of 0.702 while successfully identifying tissue patterns and genes associated with cancer survival. Since histopathological images are millions of pixels in size, they are divided into smaller patches and used as input for deep learning models. This setup where a single survival label represents hundreds or even thousands of image patches with one or more patches contributing towards the survival outcome, can be closely associated with the multiple instance learning (MIL) problem.

MIL models have been used successfully for computational pathology tasks in past literature^15–19^. A MIL-based CNN^15^ was used for prediction of patient-level survival using lung and colorectal carcinoma (CRC) histopathological images and was able to achieve state-of-the-art results with c-index of 0.6963. Another MIL-based deep learning system (DLS)^16^ was developed with model outcome significantly associated with patient survival for multiple types of cancer having a cumulative c-index of 0.61 and hazard ratio of 1.58 ($p<0.0001$). DLS achieved a 0.72 c-index and hazard ratio of 2.86 ($p=0.0034$) for a publicly available BRCA dataset. However, different from our work, the authors used a categorical approach on the survival output prediction and multiple image patches as direct input, thereby making the model highly computationally complex. Another study presented a CNN for risk categorisation with a c-index of 0.6^20^ and high/low risk classification having a hazard ratio of 2.10 ($p=0.001$) for a digital tissue microarray (TMA) dataset of breast cancer. A multi-resolution deep learning model was used in a recent study^21^ to obtain a c-index of 0.706 for a dataset of breast cancer histopathology images. However, their model required tumour, lymphocyte and nuclear segmentation maps of corresponding histopathological images to make a prediction. Apart from the methods discussed above, where images are used as input to the deep learning model, there are other approaches for survival prediction that utilize cell/tissue detection and segmentation of images using deep learning, followed by subsequent analysis of cell clusters and tissue densities^22–24^. These methods have shown promising results particularly for TNBC disease where a known relationship is available between low TILs-tumour and poor prognosis^25,26^. However, these models require time consuming and labour intensive pathologist annotations, multimodal input data, and are limited to a selected set of features derived using the given annotations.

Despite the increase in histopathology image-based deep learning methods for survival prediction in different types of cancer, such models do not show much performance improvement compared to traditional methods^20,27^ in breast cancer and TNBC disease in particular, or they require large amounts of annotations and resources which reduces scalability and reproducibility^16,19,21^. Existing MIL-based or weakly-supervised deep learning methods have not been applied to survival prediction in TNBC, potentially due to the significant challenges when analyzing histopathology images for TNBC cases. In this paper, we present our MIL-based deep learning model with attention weighted pooling for TNBC patient survival prediction from TMA images. We use a weakly labeled dataset consisting of images and patient-level information of 244 TNBC patients. We use pretrained neural networks to extract image features which are subsequently introduced as input to our MIL-based deep learning model. We also use a modified loss function to fine-tune the model after initially training it using negative partial log likelihood. We notice higher performance when using image features derived from a model pretrained on histopathological data compared to a model pretrained using ImageNet data. We also explore the effects of feature clustering and direct feature input to the model. Also, the attention heatmaps produced by our model provide explainability of the outcome through qualitative analysis by an expert pathologist. The types of tissues given higher attention by our model are related to known tissue features associated with higher risk of breast cancer-specific death, providing interpretability to the results. In addition, multivariate statistical analysis confirms that the results obtained from our model are statistically significant against routinely used clinicopathological parameters. To the best of our knowledge, this is the first deep learning study addressing survival analysis for TNBC with extensive experiments and interpretable results.

## Materials and methods

Since for each patient a single survival label represents hundreds of image patches with one or more patches contributing to the outcome, we modeled survival prediction as a deep learning-based MIL problem. Here we present the dataset and preprocessing, the feature extraction and survival prediction network architectures, the training strategy and evaluation criteria we used, and the heatmap generation for interpretation of the predictions.

### TMA dataset of TNBC patients

The TNBC dataset (Fig. 1) was acquired from St. George Hospital, Sydney, Australia (St. George Breast Boost randomised radiotherapy clinical trial^28^ NCT00138814) with clinicopathological features as previously published^29^. TMAs were constructed with sampling of $3\times 1.25$ mm cores per tumour from the periphery of each tumour as directed by a pathologist. H&E sections were digitally scanned at $40\times$ magnification (0.25 µm/pixel) using a Ventana DP200 digital scanner (Roche Diagnostics). A total of 236 patients had 3 TMA cores each, while 7 patients had 2 TMA cores each. One patient who had only 1 TMA core was removed, thereby reducing the total number of patients in our dataset to $n=243$. These TMA cores were selected from regions of interest in the whole tissue sections by an expert pathologist. The dataset contained follow-up time, overall survival status and breast cancer-specific survival status for each patient. The median follow-up was 4.3 years (range 0.02–16.3 years). There were 48 TNBC specific deaths and 71 total deaths from all causes in this cohort.

In addition to images and patient outcome, the dataset included patient-level information annotated by an experienced pathologist: tumour grade, histologic subtype, lymph node status, age at diagnosis, tumour size, TIL score (manually estimated on whole tumour sections), and TIL detections (generated through cell detection and classification built-in algorithms from QuPath^30^). A summary of the clinicopathological parameters can be found in Table 1 and a more detailed analysis of these parameters is presented in our previous work^29^. To further evaluate the performance of our model trained on the TMA dataset, we also tested it on an external set of TNBC whole slide images (WSIs) from The Cancer Genome Atlas (TCGA)^31^ public dataset.

**Table 1:** Characteristics of the clinicopathological parameters of the TNBC dataset.

Parameter	$n^*$	$\%$
**Tumour grade**		
2	12	4.9
3	231	95.1
**Tumour subtype**		
Invasive ductal carcinoma	220	90.5
Metaplastic	17	7.0
Other$$^{**}$$	6	2.5
**Lymph node positivity**		
Positive	85	35.0
Negative	155	63.8
**Age at diagnosis (median 58 years, range 18.8–91.9)**		
> 55 years	141	58.0
$\le$ 55 years	102	42.0
**Tumour size (median 22.0 mm, range 7.0–120.0)**		
> 20 mm	131	53.9
$\le$ 20 mm	112	46.1
**TIL score (median 20, range 0–90)**		
< 30	110	45.3
$\ge$ 30	133	54.7

* *n* is the number of patients in the full dataset (statistical analysis is carried out in 3 stages and the number of patients for each stage is indicated in the relevant table).

** Other includes invasive micropapillary, lobular and apocrine carcinoma.

### Data preprocessing

The dataset was preprocessed as follows. We performed a simple tissue mask generation using basic morphological operations to avoid large holes and damaged sections of the tissue cores. Firstly, the images were converted to grayscale and Otsu thresholding^32^ was applied to select valid pixels belonging to tissue areas. Next, morphological closing was performed to select all objects using an appropriate kernel. Then we retained the main tissue contour while suppressing holes larger than $\sim$1470 µm$^2$. This simple method gave us a reasonable approximation of the valid tissue masks and made it easier and more intuitive to understand the patch selection process and results. Basic geometric transformations (flips and rotations) were used to augment the dataset by up to four times ($n_{\text {aug}4\times }=972$). Additional experiments were carried out with more augmentations (flips, rotations, and colour transforms) resulting in a twelve times increase in dataset size ($n_{\text {aug}12\times }=3159$).

### Feature extraction

We used pretrained neural networks to extract features from images patches. Inspired by previous successes of using pretrained weights of deep learning models trained on natural image data^15,19,20^, we first developed a model using features derived from VGG16 pretrained on the ImageNet dataset. These models did not show competitive performance compared to state-of-the-art (Supplementary Table S1). Therefore, we experimented with neural image compression (NIC)^33,34^ which was introduced as a method of compressing giga-pixel WSIs. The NIC model uses an encoder to compress image patches of $128\times 128$ pixels into an encoding vector of size $1\times 1\times 128$. A dataset of WSIs from multiple types of cancer has been used to train the bidirectional adversarial network used in NIC. A subsequent paper by the same authors^35^ used the pretrained NIC encoder for non-small cell lung cancer subtyping to achieve state-of-the-art results.

We applied the pretrained NIC encoder to extract features from images patches. The cores were divided into non-overlapping patches of size $256\times 256$ pixels. The choice of this patch size was dependent on the pretrained model of NIC, which requires inputs of size $128\times 128$ pixels with 0.5 µm/pixel in resolution. Since the resolution of our image dataset is 0.25 µm/pixel at highest magnification, $256\times 256$ patches were downsampled by a factor of 2. This created a vector of size $1\times 1\times 128$ per patch representing a $256\times 256$ pixel area of a TMA core. Finally, patches of $512\times 512$ pixels with four adjacent non-overlapping vectors (each representing a patch size of $256\times 256$ pixels) were used as a single input. We selected patches of $512$ pixels to make sure that the tissue morphology of the selected area is represented in sufficient detail. A patient with 3 TMA cores had $\sim$220 patches of size $512\times 512$ pixels represented as $2\times 2\times 128$ vectors for the prediction pipeline.

**Figure 2:**
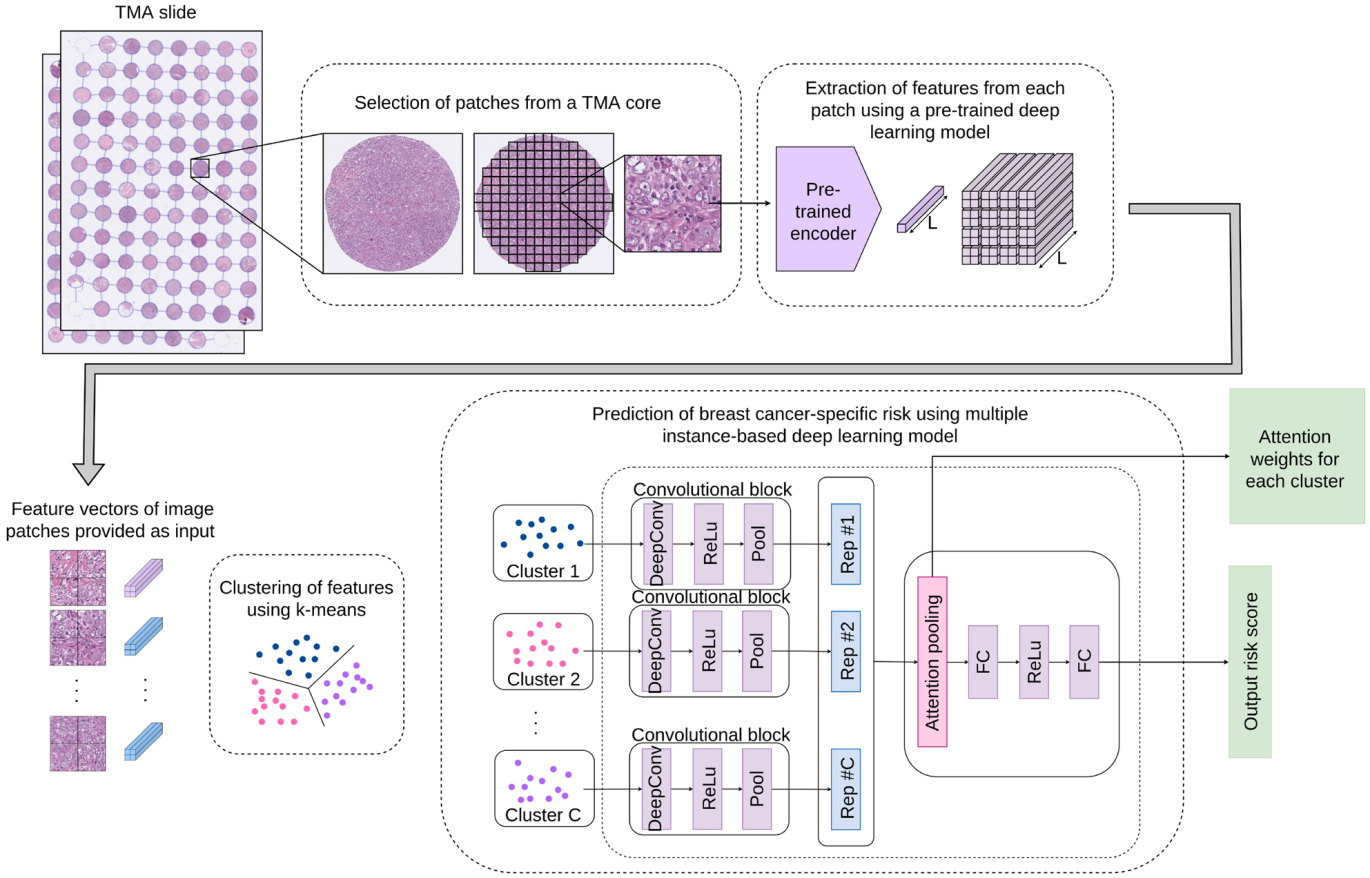
Architecture of the MIL-based survival risk prediction model using pretrained feature encodings (Model 1).

### Basic network architecture

**Figure 3:**
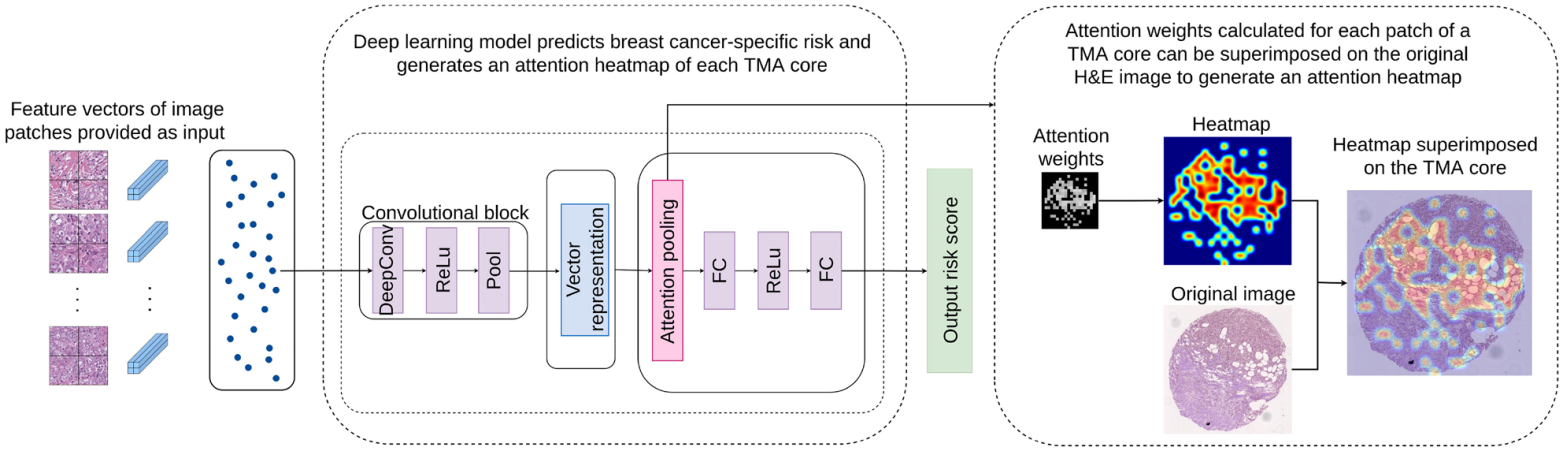
MIL-model can be reconfigured to allow each feature vector to be accepted as input to the network and attention weights be applied on each vector (Model 2). This generates an attention heatmap that highlights tissue areas of interest associated by the network as relevant to a given prediction.

Our survival prediction pipeline is inspired by^15^, where a multiple instance fully convolutional network with an attention-based representation aggregation with shared weights was used. In our experiments we considered two approaches. In the first approach (Model 1, Fig. 2), patches are clustered into phenotypes based on their deep learning features at patient-level using a *k*-means clustering algorithm. Therefore, each phenotype is a vector represented by $1\times x_i\times d$, where $x_i$ is the number of patches for the $i^\text {th}$ phenotype and *d* is the feature vector for a given patch. We experimented with several values of *k*, the details of which will be discussed later. The clustered encodings for each phenotype pass through a single layer of convolution followed by a rectified linear unit (ReLU) activation and global average pooling to produce a local representation for each phenotype cluster (dropout of 50–70% showed slight increase in performance for some experiments). For a given patient with *C* phenotype clusters, local representations can be given by $H=\{h_1, h_2, \ldots , h_C\}$ where $h_i$ is the local representation for $i^\text {th}$ phenotype. These representations are aggregated based on their attention weights according to

1 $$\begin{aligned} z=\sum _{i=1}^{C} a_i h_i, \end{aligned}$$

where *z* is the patient-level representation and $a_i$ is calculated as

2 $$\begin{aligned} a_i = \frac{\text {exp}\{w^T\text {tanh}(V h_i^T)\}}{\sum _{j=1}^{C}\text {exp}\{w^T\text {tanh}(V h_j^T)\}}, \end{aligned}$$

with $w \in \mathbb {R}^{L\times 1}$ and $V \in \mathbb {R}^{L\times M}$ being trainable parameters and *C* the number of phenotypes. Tangent $\text {tanh}(.)$ element-wise nonlinearity is used so that both positive and negative values are considered during gradient flow. This allows the model to consider similarities and dissimilarities among instances. After observing the attention weights for each phenotype cluster, we found no significant weight difference between phenotypes. Therefore in our second approach (Model 2, Fig. 3), we performed experiments with unclustered patch encodings to visualise the effect of attention weights. Here we removed the phenotype clustering step and applied the deep learning pipeline directly to the feature vector of input image patches. During the aggregation step, we calculated an attention-based weight for each patch, enabling the generation of an attention heatmap for each TMA core. By observing these results, an expert pathologist was able to identify interesting morphological tissue patterns emphasised by the model, thereby providing interpretability to the results.

### Training strategy and evaluation criteria

Similar to several related research studies^15,16,36^, we used the negative partial log likelihood as the loss function to train our model. For the $i^\text {th}$ patient, we can denote the predicted output risk score as $o_i$ and label as ($t_i$, $\delta _i$), where $t_i$ is the follow-up event time and $\delta _i$ is the censoring status. For patients whose death is not observed, $\delta _i = 0$, and for patients subjected to disease specific death, $\delta _i = 1$. Event times can be considered as an ordered set of observations where $t_1< t_2< t_3< \cdots < t_N$ for $n = N$ number of patients. For any arbitrary patient *i* whose follow-up time is $t_i$, a risk set $R(t_i)$ can be defined as the set of patients whose follow-up times are greater than or equal to $t_i$. Given that a unique event occurs at time *t*, the probability of death for patient *i* can be calculated according to $L_i$. Conditioned upon occurrence of all deaths, the joint probability of all events becomes the partial likelihood *L*.

3 $$\begin{aligned} L_i = \frac{\text {exp}(o_i)}{\sum _{j\in R(t_i)}\text {exp}(o_j)},\quad L = \prod _{i:\delta _i = 1} \frac{\text {exp}(o_i)}{\sum _{j\in R(t_i)}\text {exp}(o_j)}. \end{aligned}$$

We can maximise $\text {log}(L)$ and equivalently minimize the negative log partial likelihood over the deep learning model parameters. Therefore, the loss function for our model is defined by

4 $$\begin{aligned} L(o_i) = \sum _i\delta _i \left( -o_i + \text {log}\!\!\sum _{j:t_j\ge t_i}\!\text {exp}(o_j)\right) . \end{aligned}$$

It can be seen that $L(o_i)$ considers only the relative ordering of events when calculating the loss.

In addition to the negative log partial likelihood component, the authors of RankSurv^37^ proposed a novel loss component that takes ranking of each observation into account. Following that approach, we used a ranking-based loss component to the final loss function as a method of refining the result after the model is trained for a few epochs using negative log partial likelihood:

5 $$\begin{aligned} L_r = -\log \left( \lambda (o_i - o_j)\right) ,\quad \lambda (x) = \frac{1}{1 + \exp (-x)}. \end{aligned}$$

For optimization, we used the Adam optimizer^38^ with a weight decay of $5\times 10^{-3}$ and learning rate of $1\times 10^{-5}$ for Model 1 and a learning rate of $2\times 10^{-5}$ for Model 2. To quantify the accuracy of the predictions, we used the concordance index (c-index), which is a common evaluation criterion in survival prediction studies. For a covariate *X* and survival time *T*, assume that higher values of *X* imply shorter value for *T*. For observations 1 and 2, if $x_1 \ge x_2$ where $t_1 < t_2$, it is a pair of observations in concordance (C). If $x_1 \ge x_2$ where $t_1 > t_2$, it is a pair in discordance (D). If $x_1 = x_2$, it is an equal risk pair of observations (R). Then, concordance index ($\hat{c}$) can be defined as

6 $$\begin{aligned} \hat{c} = \frac{C+R/2}{C+D+R}. \end{aligned}$$

### Interpretability using heatmaps

To interpret the results and recognize the types of tissue morphological features and image patches that are primarily related to the result, we carried out a set of experiments with Model 2. In these experiments, feature encodings from image patches were introduced as input to the convolutional block. These encodings were aggregated based on attention weight and passed through the fully connected network to arrive at a final prediction. We carried out experiments for patch sizes of $512\times 512$ and $256\times 256$ pixels, which produced similar results. Due to the availability of greater number of data points when using interpolation of attention weights to produce the heatmap, we opted to use the $256\times 256$ patch size in this experiment.

### Ethical approval

Ethical approval was provided by the South Eastern Sydney Local Health District Human Research Ethics Committee at Prince of Wales Hospital (2018/ETH00138 and HREC 96/16), who granted a waiver of consent to perform research analyses on the tissue blocks. All methods were performed in accordance with the relevant institutional guidelines and regulations.

## Experimental results

The proposed MIL survival prediction models were trained, validated and tested on the TMA TNBC dataset and further verified against the TNBC histopathological images from the externally available public TCGA dataset. We present the details of the setup and the results obtained in the experiments with each model. Comparisons of our model performance is constrained to the different configurations of our model on internal and external data and statistical analysis of our model output against routinely used clinicopathological parameters provided by an expert pathologist. This is due to the scarcity of similar deep learning-based research with publicly available model implementations and the complexity of model development and training in order to replicate the architectures presented in past literature.

### Results of model 1

This section contains results of various experiments using Model 1 (Fig. 2).

#### Clustering experiments

We carried out several experiments with the NIC pretrained model, fine-tuned with ranking loss and randomly initialized, for $512\times 512$ image patches using different numbers of clusters, $k = 4, 6, 8, 10, 12$, generated by *k*-means unsupervised clustering algorithm, to identify if there was a relationship between the number of clusters and model performance. We performed 5-fold cross validation, using 80% of the data in the training and validation sets (divided 70/30) and 20% in the test set in each fold, and calculated the average performance for each cluster number by considering the predictions calculated for all patient through the test set of each fold (Table 2). We observed the distribution of attention weights among clusters and found that there was no significant increase in performance that can be attributed to a particular *k* value. Comparison of the average c-index shows that the effect of clustering does not affect the model performance significantly. However, much larger and smaller *k* values had comparatively lower performance. We selected $k = 10$ since it had the highest average c-index (0.616), and out of that experiment we selected the fold with the highest c-index (0.7179) for subsequent univariate and multivariate analysis. In addition, we studied the effects of adding ranking-loss to the negative partial log likelihood loss starting from different epochs of the training process (Supplementary Table S2).

**Table 2:** C-index of 5-fold cross validation results for MIL deep learning architecture (Model 1).

*k*	C-index for each fold					Average c-index
	Fold 1	Fold 2	Fold 3	Fold 4	Fold 5	
4	0.6236	0.5058	0.6213	0.6984	0.5809	0.606
6	0.5857	0.6114	0.7002	0.5624	0.5559	0.603
8	0.5717	0.5613	0.6527	0.7444	0.5306	0.612
10	0.6470	0.6118	**0.7179** $^*$	0.5764	0.5289	0.616
12	0.6494	0.4957	0.6619	0.6141	0.5931	0.603

*Trained model of the best performing fold is used for univariate and multivariate experiments.

#### Verification against TCGA TNBC dataset

We then verified our model performance against a set of TNBC cases derived from the publicly available TCGA dataset by testing them using our trained model. Out of 101 cases of TNBC, we omitted 6 cases due to unacceptably low quality of the diagnostic slides, resulting in a dataset of $n=95$ cases. The median follow-up time was 1.17 years (range 0–9.51 years). The model produced a c-index of 0.589, which proves that it can retain a reasonable level of predictive power despite high variability and poor quality of some of the WSIs. Note that due to the lower incidence of death in the TCGA dataset, our effort on trying to train using TCGA and the apply to our TMA dataset yielded a low c-index of a maximum of only 0.55.

#### Comparison of model performance with clinicopathological parameters

We selected the best performing fold from the 5-fold experiment above with highest average c-index to perform survival analysis of the results using Cox proportional hazard regression and Kaplan–Meier plots. We performed statistical analysis of our model in three stages. First, we assessed the univariate statistical significance of the model results using only the test data. However, this stage contained test data with only 10 disease-specific death events, which can be argued as an inadequate number of events. Secondly, we considered the results of our model prediction on test and validation data combined, which contains 21 disease-specific deaths. We analysed whether at this stage our model results are statistically significant in univariate and multivariate analysis against clinicopathological parameters. Finally, we applied our trained model to the entire dataset and evaluated the statistical significance of the results against clinicopathological parameters. In each case, we divided the model prediction into high/low groups based on the median value of the prediction calculated using the set of patients included in respective analysis.

*Statistical Analysis Stage 1*

**Figure 4:**
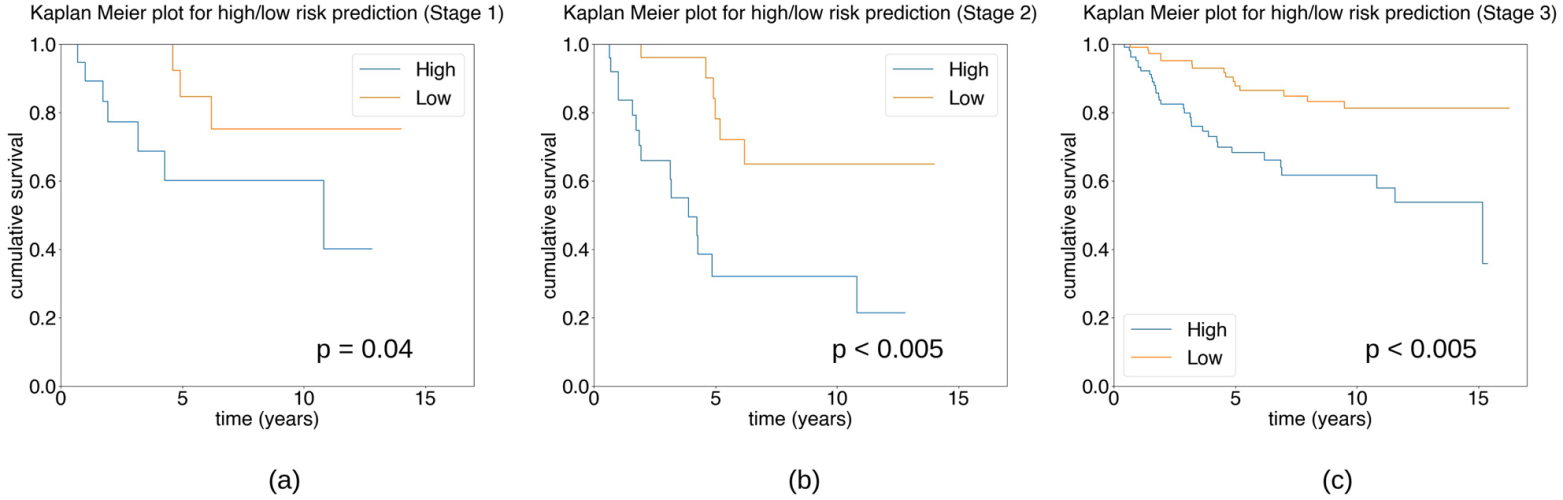
Kaplan–Meier survival estimation for three stages of analysis using the deep learning model (Model 1) output for disease-specific survival in TNBC. The plots show the results of using (**a**) only the test data (Stage 1), (**b**) test and validation data (Stage 2), and (**c**) the whole dataset of TNBC patients (Stage 3).

In the first stage, we performed statistical analysis using test data of our selected model. This included a total of 49 patients with 10 patients subject to disease-specific death. Univariate Cox proportional hazard regression for high/low risk groups with median cut-off value = 0.018 showed that the model is statistically significant: HR $=$ 4.03, 95% CI [1.04, 15.70], $p=0.04$. Figure 4a shows the Kaplan–Meier plot of this analysis stage.

*Statistical Analysis Stage 2*

In the second stage, we combined the test and validation data to perform statistical analysis of our model. This data was not used as input in the training of our model and contained 60 TNBC cases with 21 disease-specific deaths. We performed high/low risk prediction on this data set with median cut-off at 0.019. Univariate Cox proportional hazard gave statistically significant results: HR $=$ 4.77, CI 95% [1.83, 12.43], $p < 0.005$. The results of multivariate Cox proportional hazard regression are presented in Table 3. The Kaplan–Meier plot for this data is shown in Fig. 4b.

**Table 3:** Multivariate analysis for breast cancer specific survival of TNBC patients for Model 1 output on test and validation data.

Method/parameter	Cutoff value	No. of patients	Multivariate ($n=60$)		
			HR	95% CI	*p*
MIL (Model 1)	> 0.019 vs. $\le$ 0.019	30 vs. 30	4.00	1.41–11.35	0.01
Age	> 55 vs. $\le$ 55	35 vs. 25	2.69	0.98–7.37	0.05
Tumour size	> 20 vs. $\le$ 20 (mm)	36 vs. 24	4.64	1.27–16.88	0.02
LN status	pos. vs. neg.	24 vs. 36	3.23	1.18–8.82	0.02
TIL score	< 30 vs. $\ge$ 30	36 vs. 24	NS		
Grade	2 vs. 3	5 vs. 55	NS		

*NS* not significant.

*Statistical Analysis Stage 3*

In the third and final stage of our analysis, we applied the trained model to the entire dataset by dividing based on median predicted outcome with cutoff value 0.015 to categorise the patients into high- and low-risk groups. Our results for univariate and multivariate analysis of clinicopathological parameters for disease-specific survival outcome are presented in Table 4. Univariate analysis of MIL-based prediction outcome shows that it is independently statistically significant for disease-specific outcome: breast cancer specific survival HR $=$ 3.12, 95% CI [1.69, 5.75], $p<0.005$. Multivariate analysis modelling for outcome shows that the model was statistically significant accounting for standard clinicopathological parameters: model HR $=$ 2.68, 95% CI [1.44, 4.99], $p<0.005$, versus age $p=0.05$, tumour size $p=0.02$, lymph node status $p<0.005$). The Kaplan–Meier plot is shown in Fig. 4c.

**Table 4:** Univariate and multivariate analysis for breast cancer specific survival for the full dataset of TNBC patients using Model 1 output.

Method/parameter	Risk group cutoff value	No. of patients in each group	Univariate ($n=240$)			Multivariate ($n=240$)		
			HR	95% CI	*p*	HR	95% CI	*p*
MIL (Model 1)	> 0.015 vs. $\le$ 0.015	120 vs.120	3.12	1.69-5.75	< 0.005	2.68	1.44–4.99	< 0.005
Age	> 55 vs. $\le$ 55	139 vs. 101	1.98	1.08-3.62	0.03	1.87	1.00–3.49	0.05
Tumour size	> 20 vs. $\le$ 20 (mm)	129 vs. 111	2.40	1.29–4.49	0.01	2.16	1.13-4.15	0.02
LN status	pos. vs. neg.	85 vs. 155	3.21	1.80-5.71	< 0.005	2.71	1.50–4.91	< 0.005
TIL score	< 30 vs. $\ge$ 30	131 vs. 109	1.87	1.03–3.42	0.04	NS		
Grade	2 vs. 3	12 vs. 228	1.45	0.52–4.06	0.47	NS		

*NS* not significant.

These statistical tests confirm that our model is capable of outperforming some of the strongest clinicopathological parameters. Therefore, we suggest that our model could be used to elevate the effort spent by pathologists visually assessing tissue features in histopathological images. Our results provide proof of concept that our model can represent and quantitatively summate several human-derived tissue features, thereby confirming the explainability of our model.

However, it must be noted that our model does not outperform the single most significant pathological prognostic factor in breast cancer, which is lymph node status. Lymph node status is determined by pathological examination of lymph nodal sampling by sentinel node biopsy or a larger axillary dissection specimen, separate from the resection specimen containing the tumour itself. We consider that our model could prove helpful for clinicians in treatment planning and risk assessment of node negative patients to augment data available for chemotherapy decisions.

### Results of model 2

This section provides details of the results of Model 2 (Fig. 3) and the pathological implications.

#### Comparison of model performance with clinicopathological parameters

**Figure 5:**
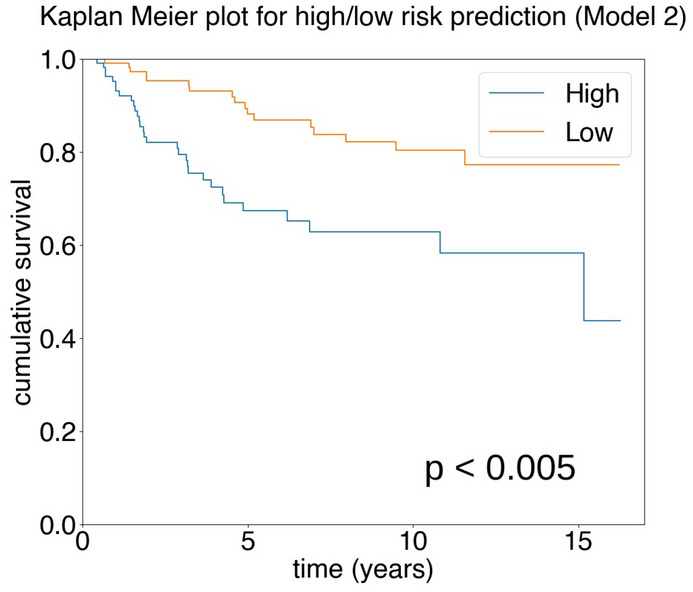
Kaplan–Meier survival estimation of high/low categories of Model 2 for disease-specific survival in TNBC.

Our experiment with Model 2 produced a c-index of 0.71 on the test dataset and 0.67 on the overall dataset. The Kaplan–Meier plot for high/low risk categorization of the results based on the median prediction for the cohort (Fig. 5) shows that the model is independently statistically significant of the outcome. The model outcome was statistically significant for univariate Cox proportional hazard regression analysis: HR $=$ 2.75, 95% CI [1.52, 4.98], $p<0.005$. From the multivariate analysis of model prediction against clinicopathological parameters using Cox proportional hazard regression (Table 5) we see that model prediction (median cutoff value = 0.21) is capable of outperforming all but the lymph node status parameter. Compared to the results from Model 1 (Fig. 4, Table 4), it can be seen that in both cases our model outputs are independently statistically significant of disease-specific outcome and statistically significant of disease-specific outcome compared to other important clinicopathological parameters.

**Table 5:** Multivariate analysis for breast cancer specific survival of TNBC patients for Model 2 output.

Parameter	Cutoff value	No. of patients	Multivariate ($n=240$)		
			HR	95% CI	*p*
MIL (Model 2)	> 0.21 vs. $\le$ 0.21	118 vs. 122	2.28	1.24–4.18	0.01
Age	> 55 vs. $\le$ 55	139 vs. 101	1.91	1.03–3.55	0.04
Tumour size	> 20 vs. $\le$ 20 (mm)	129 vs. 111	1.96	1.02–3.74	0.04
LN status	pos. vs. neg.	85 vs. 155	2.81	1.55–5.07	< 0.005
TIL score	< 30 vs. $\ge$ 30	131 vs. 109	NS		
Grade	2 vs. 3	12 vs. 228	NS		

*NS* not significant.

#### Attention heatmaps

**Figure 6:**
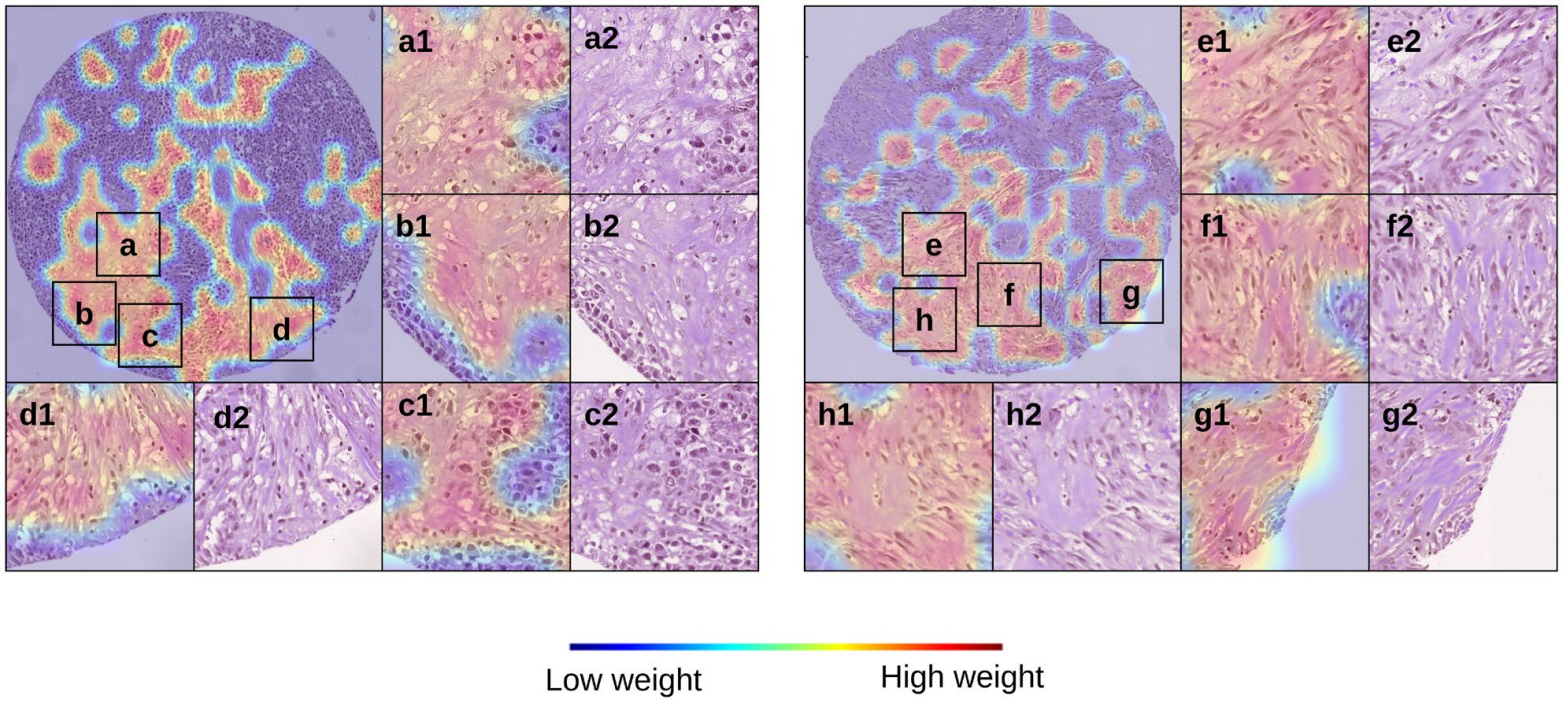
Heatmaps (a1–h1) and corresponding H&Es (a2–h2) from a representative case categorised as high-risk by the MIL classifier. The features present are those of a stroma-rich, low-TILs tumour and low-TILs tumour.

Figure 6 shows the attention heatmaps generated using the scaled attention weights (considering maximum and minimum weights of the total dataset). By observing the attention-heatmaps from the experiments with Model 2, we were able to recognize key morphological characteristics appearing repeatedly in patients with high-risk predictions (high/low risk groups categorized using the median risk prediction). The most prominent histological features that have been identified by a pathologist through qualitative analysis of our results are: high-grade carcinoma/tumour epithelium, high stromal content, infiltrative growth pattern, and low TILs. As indicated in Table 4 and previous studies^22,23,25,29^ low TILs-tumours have been shown to have poorer survival and poorer response to chemotherapy compared to high TILs TNBC tumours. Our model prediction is consistent with this observation where cases assigned high-risk correspond to those tumours with low levels of infiltrating TILs, indicating low levels of anti-tumour immunity (so called “immune cold” tumours). Although high stromal content and infiltrative growth patterns are not currently used as standard pathological features, there is evidence that tumour stromal ratio (TSR) is prognostic in previous published work in this cohort^29^ and other independent studies^39,40^. Scoring of TILs and stromal content is difficult for human observers, and computational tools may be a more reproducible way of assessing these features. Our model also detects carcinoma epithelium, which may reflect subtle morphologic cellular changes that potentially correspond with an underlying molecular phenotype associated with risk. However, we do not have any genomic characterisation as yet to further assess these possible associated genomic features. Supplementary Fig. S1 shows more heatmaps of TMA cores with similar observations for stroma-rich, low-TILs areas of tumour highlighted by our model.

## Discussion

This study aimed to create a deep learning model to predict survival outcome in triple negative breast cancer patients using histopathological images and patient-level information. We have demonstrated that our deep learning model has state-of-the-art performance with interpretable results, which correspond to known high-risk features in TNBC. Interestingly, our MIL model appears to capture these important features with a unified risk prediction score of independent statistical significance which is of clinical relevance for highly aggressive TNBC. As most patients will be offered adjuvant chemotherapy to reduce the risk of recurrent disease, there may be a clinical scenario where chemotherapy (and hence unwanted toxicities) may want to be avoided for a MIL low-risk, node negative patient.

Although our model presents compelling results, there are several limitations and improvements that can increase clinical applicability of our method. In particular, our dataset was acquired from one institution, which uses relatively uniform and standardised tissue processing and staining protocols. Also, compared to using WSIs, our image data captures smaller areas of TMA cores extracted from whole tissue sections. Since our model achieves good results despite the constraints on the diversity of tissue morphologies captured in image patches, we expect that adapting our method for larger datasets of WSIs would produce better performance. Therefore, future research will be carried out on development and testing of deep learning models using more data from multiple institutions with more variation in tissue fixation and H&E staining that can improve performance, highlight prognostic signals, and increase applicability to clinical practice in divergent geographical locations. Moreover, in this study, we have trained our model using weakly labeled data. Our work can be extended using additional manual annotations such as tissue region annotations and cell annotations (such as immune cell annotations) that have the potential of improving performance and interpretability of survival prediction outcome. Such manual annotations can be used for quantitative evaluation of the tissue structures as opposed to the current qualitative approach. Finally, one of our key findings from the statistical analysis using clinicopathological parameters is the importance of lymph node status for the survival prediction task. Since our dataset contains only images extracted from tumour regions of whole tissue sections, a promising future area of improvement would be to incorporate additional specimens such as lymph nodes sections.

TSR is emerging as a prominent feature in tumour biology and prognostic significance. We and others^29,39,40^ have consistently shown that TNBC tumours with abundant stroma (the supportive tissue containing collagen, fibroblasts, immune cells and blood vessels within which the tumour cells reside) have a poorer prognosis compared to stroma-poor TNBC. Both TILs density (scored as a percentage of the peri-tumoural stroma occupied by immune cells) and TSR are difficult to be visually quantified by expert pathologists accurately and reproducibly, suffering from much subjective interpretation. Thus, there have been recent attempts to more accurately quantify these parameters using deep learning approaches^13,41–43^ in many tumour types to determine their prognostic significance as individual prognostic factors. One other tumour feature identified in the heatmaps of TNBC disease-specific deaths for high-risk cases was infiltration of fat by the tumour. The basis for this finding is currently uncertain in terms of biology as it is commonly observed in routine clincial pathology practice, but will be further assessed in ongoing work. It is noted that a similar risk association for fat infiltration was found in a deep learning survival prediction study in colorectal carcinoma^44^. Supplementary Fig. S2 shows examples of heatmaps with fat infiltrates assigned higher attention weight by our model.

These findings support the explainability of our AI predicted tumour features, which is critical to further enhance validity and develop trust amongst clinicians regarding the application of deep learning tools to clinical pathology and oncology practice. This study demonstrates proof of principle that our system has potential as a clinical decision assisting tool, subject to further development, validation and testing in other independent datasets.

## Supplementary Information

Supplementary Information.

## Data Availability

The TNBC TMA image dataset and clinical data are not publicly available due to ethical restrictions but may be accessible on reasonable request to the corresponding author. TCGA image data and survival data are available publicly through https://portal.gdc.cancer.gov/.
