# Supplementary material for: Survival prediction in triple negative breast cancer using multiple instance learning of histopathological images
Source: Sci Rep. 2022 Aug 25;12:14527. doi: 10.1038/s41598-022-18647-1 (PMC9411153; doi:10.1038/s41598-022-18647-1)
Supplement: Supplementary file 1 — Supplementary Information. [file 41598_2022_18647_MOESM1_ESM.pdf]

Survival Prediction in Triple Negative Breast Cancer Using Multiple Instance Learning of Histopathological Images: Supplementary Materials

Piumi Sandarenu, Ewan K. A. Millar, Yang Song, Lois Browne, Julia Beretov, Jodi Lynch, Peter H. Graham, Jitendra Jonnagaddala, Nicholas Hawkins, Junzhou Huang, Erik Meijering

Table S1: C-index of 5-fold cross validation results on TNBC data using feature encodings derived from VGG16 pre-trained model.

No. clusters	c-index for each fold					Average c-index
	1	2	3	4	5	
10	0.5184	0.5353	0.5312	0.5615	0.5951	0.5483
12	0.4865	0.5279	0.6719	0.4581	0.6113	0.5511

Table S2: C-index of 5-fold cross validation results on TNBC data using feature encodings derived from NIC pre-trained model for ranking-loss added from different epochs of training.

No. clusters	Loss[a] (epoch)	c-index for each fold					Average c-index
		1	2	3	4	5	
10	35	0.6470	0.6118	0.7179[b]	0.5746	0.5289	0.6160
10	40	0.6189	0.6511	0.7078	0.5519	0.5196	0.6099
10	0	0.6007	0.6447	0.6772	0.5137	0.5117	0.5890

[a] Ranking loss component added to negative log partial likelihood loss from given epoch onwards.

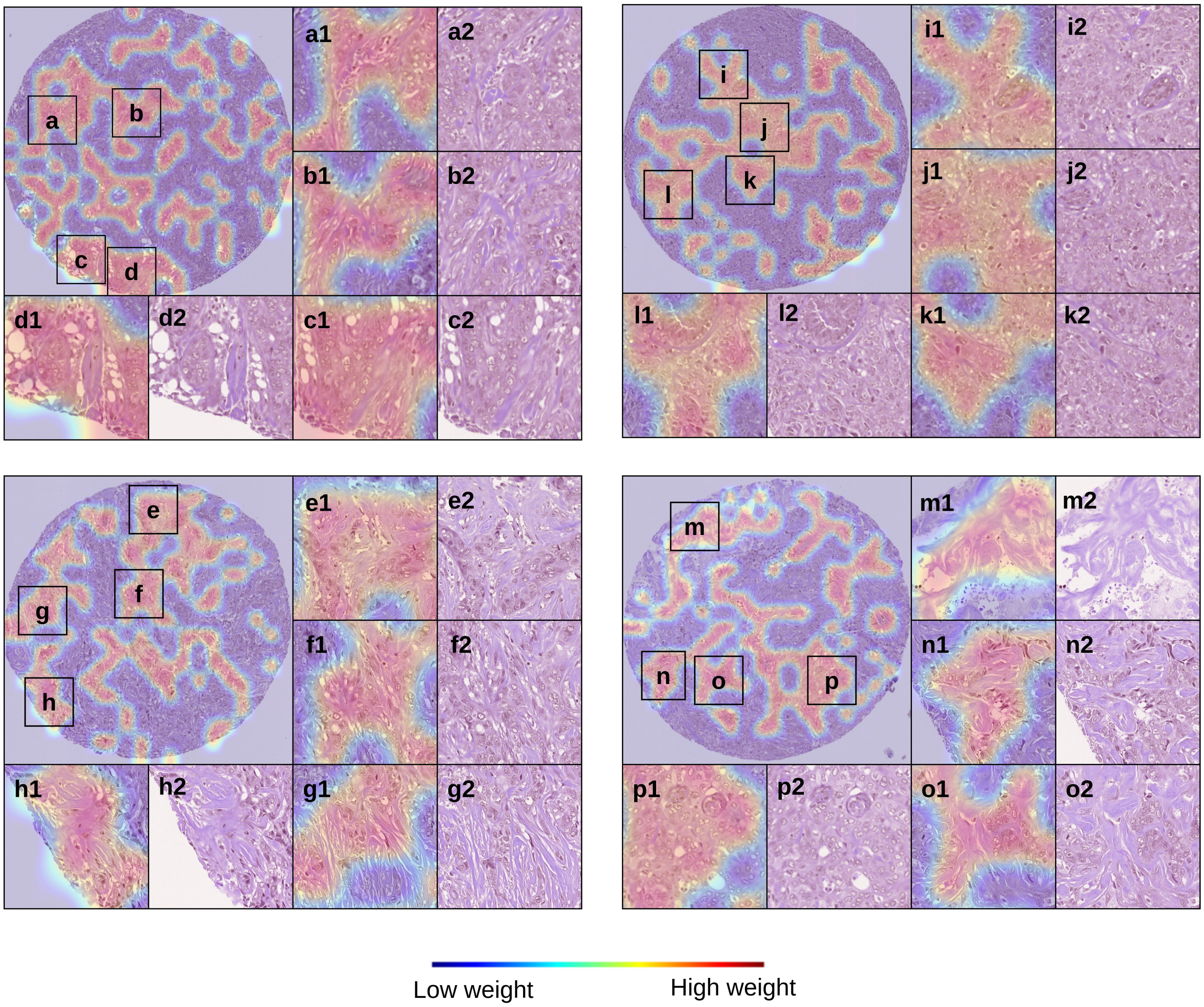

Figure S1: Heatmaps (a1-p1) and corresponding H&Es (a2-p2) from a representative case categorised as high-risk by the MIL classifier. The features present are those of a stroma-rich, low-TILs tumour and low-TILs tumour.

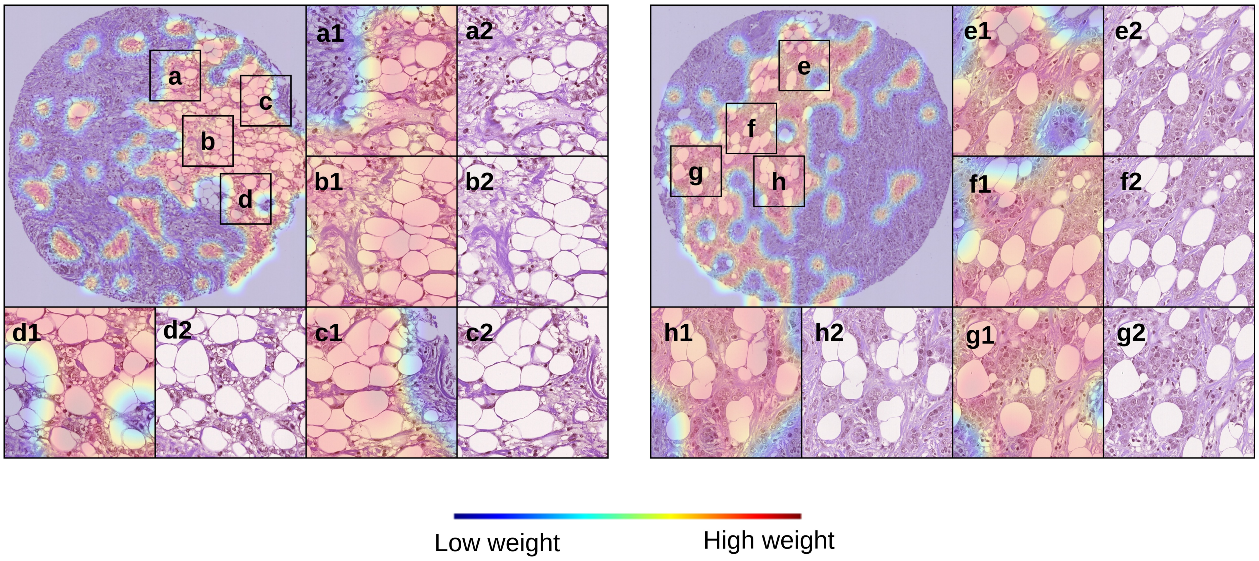

Figure S2: Heatmaps (a1-h1) and corresponding H&Es (a2-h2) from a representative case categorised as high-risk by the MIL classifier. The features present are those of low-TILs tumour with infiltration of fat by cancer cells.
